# The role of single nucleotide polymorphisms of *IL-1A *-889C>T (rs1800587), *TNF-A *-238G>A (rs361525), and *VDR TaqI* (rs731236) on susceptibility to herniated nucleus pulposus

**DOI:** 10.12688/f1000research.53235.1

**Published:** 2021-05-25

**Authors:** Azharuddin Azharuddin, Muhammad Ilmawan, Jonny Karunia Fajar, Marhami Fahriani, Sukamto S. Mamada, Helnida Anggun Maliga, Firzan Nainu, Kuldeep Dhama, Harapan Harapan, Rahadyan Magetsari

**Affiliations:** 1 Department of Orthopedic and Traumatology, School of Medicine, Universitas Syiah Kuala, Banda Aceh, Aceh, 23111, Indonesia; 2 Department of Orthopedic and Traumatology, Dr. Zainoel Abidin Hospital, Banda Aceh, Aceh, 24415, Indonesia; 3 Faculty of Medicine, Universitas Brawijaya, Malang, East Java, 65117, Indonesia; 4 Medical Research Unit, School of Medicine, Universitas Syiah Kuala, Banda Aceh, Aceh, 23111, Indonesia; 5 Brawijaya Internal Medicine Research Center, Department of Internal Medicine, Faculty of Medicine, Universitas Brawijaya, Malang, East Java, 65145, Indonesia; 6 Faculty of Pharmacy, Hasanuddin University, Tamalanrea, Makassar, South Sulawesi, 90245, Indonesia; 7 Division of Pathology, ICAR-Indian Veterinary Research Institute, Izatnagar, Bareilly, Uttar Pradesh, 243122, India; 8 Orthopaedic and Traumatology Division, Department of Surgery, Faculty of Medicine, Universitas Gadjah Mada, Yogyakarta, DKI Yogyakarta, 55281, Indonesia; 9 Orthopaedic and Traumatology Division, Department of Surgery, Dr. Sardjito Hospital, Yogyakarta, DKI Yogyakarta, 55281, Indonesia

**Keywords:** Spinal disc herniation, SPN, IL-1A, TNF-A, VDR

## Abstract

**Background**: The objective of this study was to determine the role of single nucleotide polymorphisms (SNPs) in interleukin 1 alpha ( *IL-1A*), tumor necrosis factor-alpha ( *TNF-A*), and vitamin D receptor ( *VDR*) genes on the susceptibility to herniated nucleus pulposus (HNP).

**Methods**: Four databases (PubMed, Embase, Cochrane, and Web of Science) were searched as of April 1 ^st^, 2021. Authors, publication year, targeted genes, genotype and allele frequency in each case and control groups were collected. Newcastle-Ottawa scale was used to evaluate the publication quality. The pooled estimates of association of *IL-1A *-889C>T (rs1800587), *TNF-A *-238G>A (rs361525), and *VDR TaqI* (rs731236) and susceptibility to HNP were assessed using Z test and presented as odd ratio (OR) and 95% confidence intervals (95%CI).

**Results**: We screened 3,067 unique studies for eligibility and three, two and nine studies on *IL-1A *-889C>T, *TNF-A *-238G>A, and *VDR TaqI *were included, respectively, in our meta-analysis. The studies consisting 369 HNP cases and 433 controls for *IL-1A *-889C>T, 252 cases and 259 controls for *TNF-A *-238G>A and 1130 cases and 2096 controls for *VDR TaqI. *Our pooled estimates indicated that there was no significant association of those SNPs with the susceptibility to HNP in any genotype, dominant model, recessive model, or allele comparations.

**Conclusion**: Although individual studies suggested the important role of gene expression dysregulation associated with SNPs in *IL-1A*, *TNF-A*, and *VDR*, our data indicated that *IL-1A *-889C>T, *TNF-A *-238G>A, and *VDR TaqI *had weak association with HNP susceptibility in both genotypes and allele distributions. However, since heterogeneity was identified among studies included in this meta-analysis, further meta-analysis with a larger population and subgroup analysis on specific population are warranted to support this finding.

## Introduction

Herniated nucleus pulposus (HNP) or disc herniation is the most common spinal degenerative disease associated with lower back pain and radicular pain of the lower extremities due to nerve compression. ^1^ HNP is also the most common cause of persistent sciatic pain due to displacement of the nucleus pulposus beyond the intravertebral disc space. ^2^ The most prevalent HNP locations are between the L4 and L5 vertebrae and between the L5 and S1 vertebrae ^3^ whilst the highest incidence is observed amongst people aged 30-50 years old. ^4^ Diabetes, ^5^ smoking, ^5, 6^ obesity, ^5- 7^ type of occupation, ^8^ age, ^7^ and gender ^4, 9^ have all been associated with a high risk of developing disc degenerative diseases. However, it has been suggested that genetic factors also play a vital role in susceptibility to disc degenerative diseases. A study showed that individuals aged younger than 30 years who have a family history of disc herniation have a 14.5 times higher risk of developing disc protrusion than individuals who have no family history. ^8^ Family history is also attributed to a 5.1 times higher risk of disc herniation in people aged between 30-50 years old. ^8^ The Twin Spine Study found that heredity substantially influences disc degeneration by 43-77%. ^10, 11^

The intervertebral disc (IVD) consists of two different components: the nucleus pulposus (NP) and the annulus fibrosus (AF), ^12^ where proteoglycans (mostly found in NP) acts as an internal semi-fluid mass and collagen (mostly found in AF) acts as a laminar fibrous container. ^13^ Genes encoding components of IVD such as collagens I, ^14^ collagens IX, ^15^ collagens XI, ^16^ aggrecan, ^17^ cartilage intermediate layer protein (CILP), ^18^ and vitamin D receptor (VDR) ^19^ have previously been studied to determine susceptibility to lumbar disc diseases. Other factors such as increased production of extracellular matrix-degrading enzymes (encoded by matrix metalloproteinase 3 gene ( *MMP-*3) and *MMP-9* ^20^ and increased expression of inflammatory cytokines such as interleukin-1 alpha (IL-1A), IL-18, ^21^ IL-6, and tumor necrosis factor-alpha (TNF-α) ^22^) are commonly found in disc degeneration. Excessive synthesis, secretion, and biological activity of these inflammatory mediators are associated with tissue destruction and are therefore commonly found in inflammatory disorders including disc degeneration. ^23^

One of the mechanisms that alters the production of protein mediators in the human body are single-nucleotide polymorphisms (SNPs). These genetic variations, single nucleotide changes at specific positions in a gene, may influence gene expression and hence associate to particular disease. A three-fold increase in susceptibility of disc degeneration was observed in individuals with a TT genotype compared to those without the allele (CC genotype) on SNP *IL-1A* -889C>T (rs1800587). ^24^ People with minor allele of *IL-1A* -889C>T (T allele) also had a 2.4-fold increased risk of disc bulges ^24^ and a 2.5-fold increased risk of endplate modic change. ^25^ A study in an Iranian population found that among nine SNPs on pro-inflammatory cytokine genes ( *IL-1*, *IL-6* and *TNF-A*), no association to IVD degeneration was found except for two SNPs in the *TNF-A* gene ( *TNF-A*−308 G/A and *TNF-A* −238 G/A). ^19^ TNF-α plays important role in the pathophysiology of HNP such as upregulating the activity and the gene expression of MMP, stimulating other cytokines such as IL-1, IL-6, and IL-8, stimulating cell migration, altering endothelial permeability, and decreasing the synthesis of collagen and proteoglycan. ^26^ A study reported that G allele and GG genotype of *TNF-A* 238G>A (rs361525) were 2.51 times and 2.98 times, respectively, more prevalent in patients with HNP compared to healthy controls. ^19^

Several roles of VDR such as regulating chondrocyte proliferation and differentiation, bone mineralization and remodeling, and matrix production have previously been demonstrated. ^27^ VDR's role in spinal degenerative disorder has been studied in Italian, ^28^ Turkish, ^29^ and Southern European populations. ^30^ A study in a Chinese population suggested that subjects with the t allele of *VDR TaqI* (rs731236) had a 2.61 times higher risk to have degenerative disc disease. ^31^ Moreover, individuals aged younger than 40 years who had the t allele were almost six times more likely to develop disc degeneration and 7.17 times more likely to develop disc bulge compared to those without the t allele. ^31^ However, studies in Danish ^32^ and Mexican populations ^33^ contradict previous results suggesting a role of *VDR TaqI* in disc degenerative disease. This conflicting role of SNPs in *IL-1A, TNF-A* and *VDR* on HNP therefore needs to be further evaluated. This study sought to determine the association of *IL-1A* -889C>T (rs1800587), *TNF-A* 238G>A (rs361525), and *VDR TaqI* (rs731236) in susceptibility to HNP.

## Methods

### Study design and protocol

A systematic review and meta-analysis were conducted to assess the association of three SNPs, *IL-1A* (rs1800587), *TNF-A* (rs361525), and *VDR* (rs731236), on susceptibility to HNP. The outcome variable of this study was the risk or susceptivity to have HNP while the response variables were the SNPs in three genes: *IL-1A* (rs1800587), *TNF-A* (rs361525), and *VDR* (rs731236). We searched databases for relevant studies, then extracted and analyzed data from those studies to achieve the pooled odds ratios (ORs) and 95% confidence interval (95%CI) using a random or fixed effect model depending on the data. This study was conducted in accordance with the Preferred Reporting Items for Systematic Reviews and Meta-analyses (PRISMA) guideline. ^34, 35^ The protocol of this study has been registered in PROSPERO (reg. number CRD42021249187).

### Literature search strategy

The literature searches were conducted on PubMed, Embase, Cochrane, and Web of Science. The searches were conducted using the keywords:‘degenerative disc disease’ AND (‘IL-1A’ OR ‘rs1800587’ OR ‘-889C>T’) OR (‘TNF-A’ OR ‘rs361525’ OR ‘-238G>A’) OR (‘VDR’ OR ‘rs731236’ OR ‘TaqI’) AND ‘gene polymorphism’, including all results up to April 1 ^st^, 2021. The keywords were adapted from Medical Subject Heading (MeSH). Additional studies were also retrieved from the references of relevant papers. If two or more studies with the same study data were identified, the most recent study was used. The processes were conducted by three independent authors (JKF, MI, HAM).

### Study eligibility

To be eligible for the meta-analysis, a study had to meet all the inclusion criteria below: ( 1) the study design should be case-control, cross-sectional, or cohort design; ( 2) the study should evaluate the association of *IL-1* (rs1800587), *TNF-A* (rs361525), or *VDR* (rs731236) on HNP and have case and control groups; and ( 3) studies should present genotype frequency or minor allele frequency (MAF). All studies with duplicate records, poor quality or which had deviation from Hardy-Weinberg Equilibrium (HWE) were excluded. ^36^

### Data extraction

Important information from the studies such as first author name, year of publication, names of targeted gene and the SNP, genotype frequency, or MAF from case and control groups were collected. The allele frequency and MAF were recalculated using Mendel’s law. Data extraction processes were conducted by three independent authors (JKF, MI, HAM) and consensus established together with senior authors (AA, HH) if discrepancies were found.

### Quality assessment

The quality of the included studies was evaluated using Newcastle-Ottawa Score (NOS) ^37^ by three independent authors (JKF, MI, HAM). This evaluation was conducted to ensure the quality of three fundamental methodological parameters of the studies: patient selection (four points), comparability of the groups (two points), and ascertainment of exposure (three points); NOS ranged from 0 to 9. Each study was then categorized based on the NOS: (1) good quality (NOS ≥ 7); (2) moderate quality (NOS ≥ 5); or (3) poor quality (NOS < 5). Consensus was established if discrepancies were found.

### Statistical analysis

To assess the association of *IL-1A* (rs1800587), *TNF-A* (rs361525), and *VDR* (rs731236) on HNP, a Z-test was employed. The Egger test was used to evaluate the publication bias and a p < 0.05 indicated the possibility of publication bias in each calculated result. The Q test was used to evaluate the heterogeneity and decide between random and fixed-effect models for OR calculation. If heterogeneity was indicated (p-value less than 0.10), the random effect model was used; otherwise, the fixed-effect model was used. All analyses were performed using ‘meta’, ^38^ ’ metafor’, ^39^ and ‘dmetar’ ^40^ packages in R version 4.0.4. ^41^

## Results

### Study eligibility results

**Figure 1.**
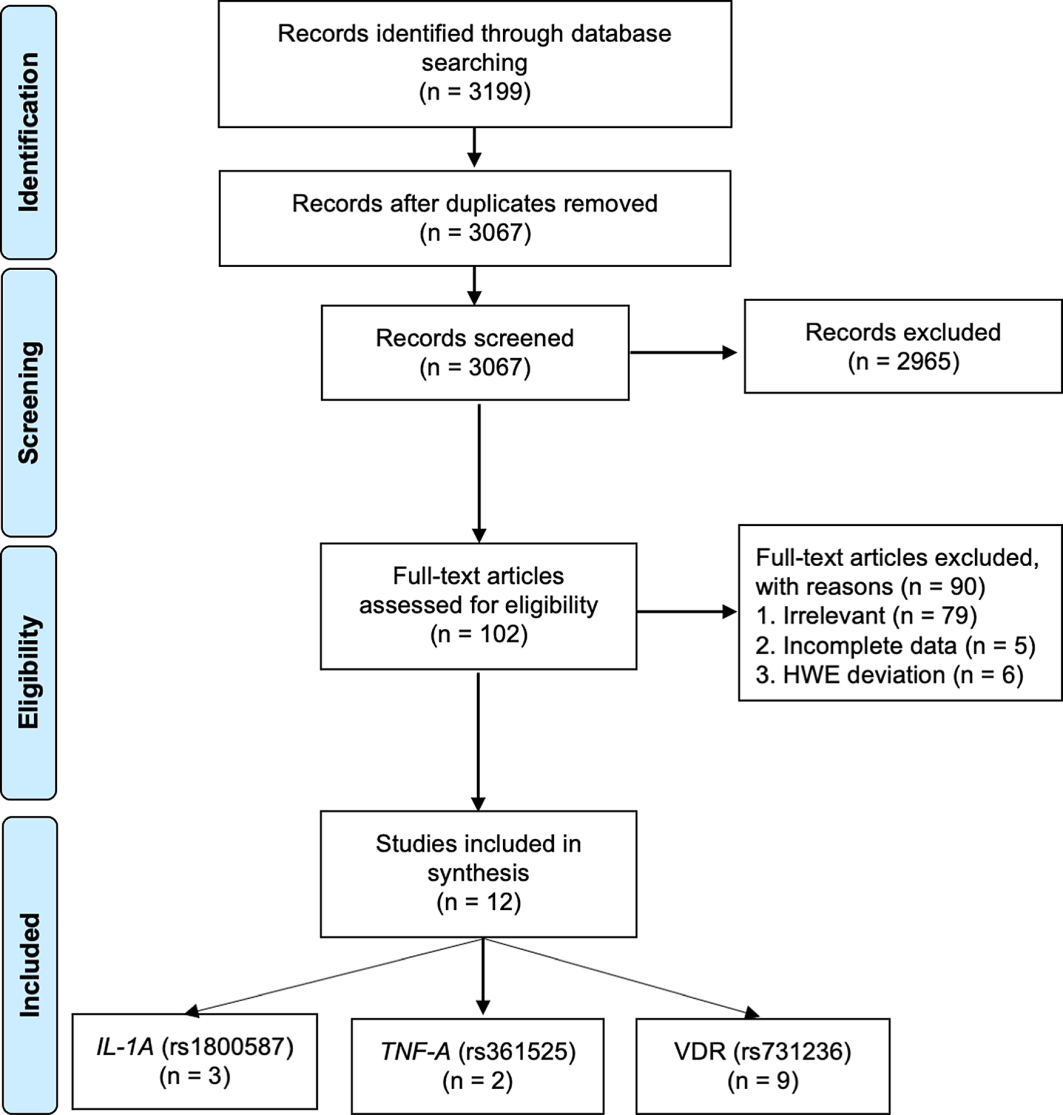
Flowchart of the result of literature searches according to the preferred reporting items of systematic reviews and meta-analyses (PRISMA).

The literature searches yielded 3,199 articles of which 3,067 references were retained after removing duplicates. Screening of the titles and abstracts excluded 2,965 articles as they did not meet the inclusion criteria. After a further screening of full text, an additional 90 studies were excluded due to lack of relevance (n = 90), incomplete data (n = 5), and HWE deviation (n = 6) ( Figure 1). 12 studies were included in the meta-analysis: three studies for *IL-1A* (rs1800587), ^19, 42, 43^ two studies for *TNF-A* (rs361525), ^19, 44^ and nine studies for *VDR* (rs731236). ^28, 42, 45- 51^ The summary of studies included in the meta-analysis is presented in Table 1.

**Table 1.** Study characteristics of *IL-1A* -889C>T, *TNF-A* 238G>A, and *VDR TaqI* (rs731236) on HNP.

Gene	SNP code	Author	Year	Country	Study design	NOS	Study groups															
							Case								Control							
							Genotype				Allele			HWE	Genotype				Allele			HWE
							AA	AB	BB	*n*	A	B	*n*		AA	AB	BB	*n*	A	B	*n*	
*IL1A*	-889C>T						CC	CT	TT		C	T			CC	CT	TT		C	T		
		Serrano et al ^42^	2014	Mexico	Case-control	8	51	45	4	100	147	53	200	2.41	55	35	10	100	145	55	200	1.49
		Chen et al ^43^	2018	China	Case-control	8	87	78	28	193	252	134	386	2.27	102	81	14	197	285	109	394	0.15
		Abdollahzade et al ^19^	2018	Iran	Case-control	8	33	33	10	76	99	53	152	0.15	62	62	12	136	186	86	272	0.40
*TNF-A*	238G>A						GG	GA	AA		G	A		GG	GA	AA		G	A			
		Aparicio et al ^44^	2011	Spain	Case-control	7	45	5	0	50	95	5	100	0.14	113	9	0	122	235	9	244	0.18
		Abdollahzade et al ^19^	2018	Iran	Case-control	8	61	15	0	76	137	15	152	0.91	79	57	1	137	215	59	274	7.32
*VDR*	*TaqI*						TT	TC	CC		T	C			TT	TC	CC		T	C		
		Chen et al ^45^	2012	China	Case-control	8	79	2	0	81	160	2	162	0.01	86	14	1	101	186	16	202	0.25
		Cheung et al ^46^	2012	China	Case-control	9	92	15	1	108	199	17	216	0.19	103	3	0	106	209	3	212	0.02
		Colombini et al ^28^	2016	Italy	Case-control	8	114	117	35	266	345	187	532	0.33	106	109	37	252	321	183	504	1.06
		Eser et al ^47^	2010	Turkey	Case-control	8	65	67	17	150	198	102	300	0.00	67	66	16	150	201	99	300	0.00
		Li et al ^48^	2018	China	Case-control	8	114	6	0	114	234	6	240	0.08	109	11	0	120	229	11	240	0.28
		Oishi et al ^49^	2003	Japan	Case-control	8	31	8	0	39	70	8	78	0.51	16	5	0	21	37	5	42	0.38
		Omair et al ^50^	2012	Norway	Case-control	9	53	70	23	147	176	116	292	0.00	61	92	35	188	214	163	376	0.00
		Serrano et al ^42^	2014	Mexico	Case-control	8	69	27	4	69	165	35	200	0.42	62	35	3	100	159	41	200	0.54
		Yuan et al ^51^	2010	China	Case-control	8	156	22	0	156	334	22	356	0.77	256	28	0	284	540	28	568	0.76

HWE: Hardy-Weinberg Equilibrium *, IL-1A*: interleukin 1A gene, NOS: New Castle Ottawa Scale *, p-Het*: p-heterogeneity, *TNF-A:* tumor necrosis factor alpha gene, *VDR*: vitamin D receptor gene.

### Distribution of allele and genotype frequency of *IL-1A* -889C>T, *TNF-A* 238G>A and *VDR TaqI*

Our data indicated that the TT genotype of *IL-1A -889C>T* was 1.37 more frequent in HNP patients than in controls, while the distribution of alleles and other genotypes were similar between patients and healthy controls. CT genotype and T allele of *TNF-A* 238G>A were both 1.6 times more frequent in healthy controls than in HNP cases. No difference in the distribution of alleles or genotypes between HNP and controls was observed in *VDR TaqI* **(** Table 1 **)**.

### Association between alleles and genotypes of *IL-1A*, *TNF-A*, and *VDR* polymorphism and HNP

**Figure 2.**
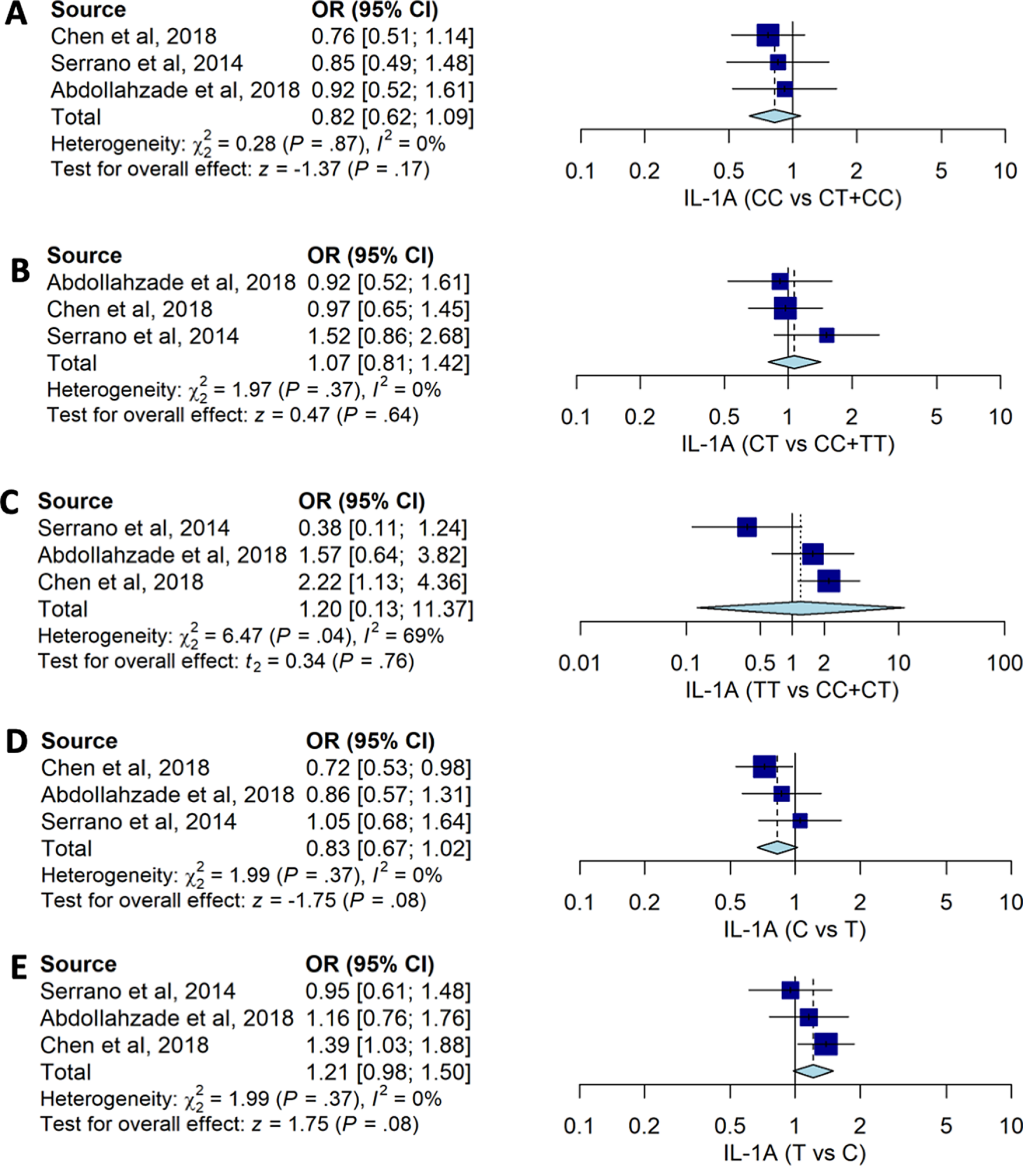
The forest plot of the association of *IL-1A*-889C>T and HNP. (A) CC vs CT+TT (OR: 0.82; 95%CI: 0.62, 1.09); p-value0.170, p-Het 0.867, and p-Egger 0.193, (B) CT vs CC+TT (OR1.07; CI95%: 0.81, 1.42); p-value 0.637; p-Het 0.373; p-Egger 0.675, (C) TT vs CT+CC (OR: 1.20; CI95%: 0.13, 11.37); p-value 0.764, p-Het 0.039, p-Egger 0.177, (D) C vs T (OR0.83; 95%CI: 0.67, 1.02); p-value 0.081; p-Het 0.369; p-Egger 0.221), and (E) T vs C (OR: 1.21; CI95%: 0.98, 1.50); p-value 0.081; p-Het 0.369; p-Egger 0.221.

Our pooled estimates suggested that no *IL-1A* -889C>T genotypes were associated with the risk of HNPs with CC vs. CT+TT (OR: 0.82, 95%CI: 0.62, 1.09), CT vs. CC+TT (OR: 0.07; 95%CI: 0.81, 1.42), and TT vs. CT+CC (OR: 1.20; 95%CI: 0.13, 11.37) ( Table 2 and Figure 2). The pooled data also suggested that allele frequency of *IL-1A* -889C>T had no significant association with the susceptibility to HNP with OR: 0.83; 95%CI: 0.67, 1.02 for C allele compared to T allele.

**Table 2.** Associations of genotypes and alleles of *IL-1A* -889C>T, *TNF-A* 238G>A, and *VDR TaqI* (rs731236) on HNP.

Gene	Allele/genotype model	Number of studies	Model	OR (CI 95%)	p-value	p-Het	p-Egger
*IL-1A*	CC *vs.* CT+TT	3	Fixed	0.82 (0.62, 1.09)	0.170	0.867	0.193
	CT *vs.* CC+TT	3	Fixed	1.07 (0.81, 1.42)	0.637	0.373	0.675
	TT *vs.* CT+CC	3	Random	1.20 (0.13, 11.37)	0.764	0.039	0.177
	C *vs.* T	3	Fixed	0.83 (0.67, 1.02)	0.081	0.369	0.221
	T *vs.* C	3	Fixed	1.21 (0.98, 1.50)	0.081	0.369	0.221
*TNF-A*	GG *vs.* GA+AA	2	Random	1.60 (0.00,12882.74)	0.628	0.034	0.889
	GA *vs.* GG+AA	2	Random	0.63 (0.00, 4211.31)	0.629	0.038	0.864
	AA *vs.* GG+GA	2	Random	1.34 (0.16, 6712.96)	0.955	0.587	<0.001
	G *vs.* A	2	Random	1.60 (0.00, 12882.74)	0.628	0.034	0.744
	A *vs.* G	2	Random	0.67 (0.00, 1555.67)	0.629	0.056	0.744
*VDR*	TT *vs.* TC+CC	9	Random	2.65 (0.60, 11.85)	0.172	<0.001	0.986
	TC *vs.* TT+CC	9	Random	1.01 (0.55, 1.85)	0.964	0.041	0.791
	CC *vs.* TC+TT	9	Fixed	0.94 (0.69, 1.28)	0.688	0.970	0.3871
	T *vs.* C	9	Random	1.06 (0.91, 1.22)	0.775	0.023	0.874
	C *vs.* T	9	Random	0.92 (0.50, 1.72)	0.775	0.023	0.874

*IL-1A*: interleukin 1A gene, *p-Het*:p-heterogeneity, *TNF-A:* tumor necrosis factor alpha gene, *VDR*: vitamin Dreceptor gene.

**Figure 3.**
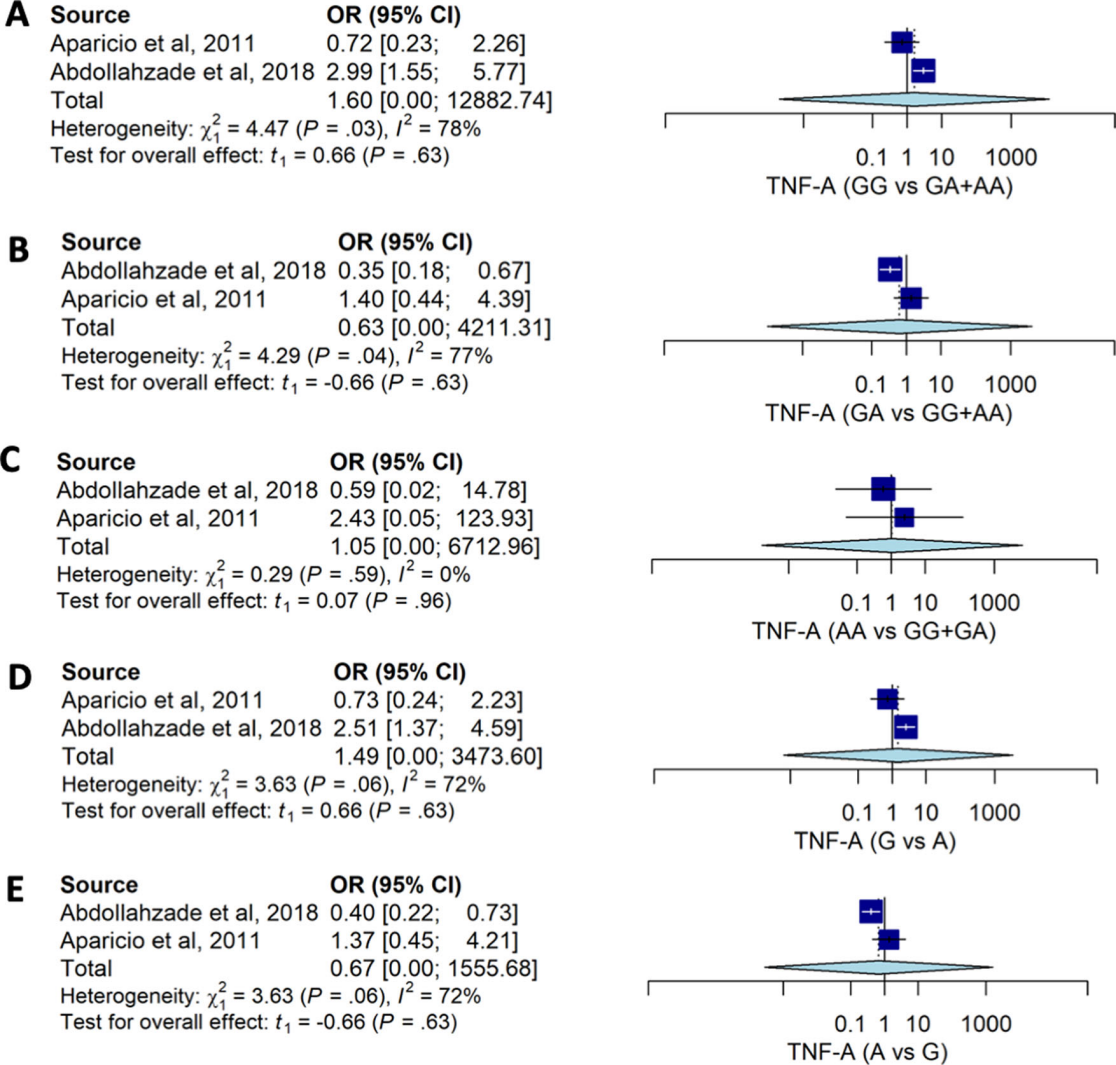
The forest plot of the association of *TNF-A* 238G>A and HNP. (A) GG vs GA+AA (OR1.60; CI95%: 0.00, 12882.74); p-value 0.628, p-Het 0.034, p-Egger 0.889, (B) GA vs GG+AA (OR0.63; CI95%: 0.00, 4211.31); p-value 0.629, p-Het 0.038, p-Egger 0.864, (C) AA vs GG+GA (OR1.05; CI95%: 0.16, 6712.96); p-value 0.955, p-Het 0.587, p-Egger <0.001, (D) G vs A (OR1.49; 95%CI: 0.00, 3473.60); p-value 0.629, p-Het 0.034, p-Egger 0.744, (E) A vs G (OR: 0.67; 95%CI:0.00, 1555.67); p-value 0.629, p-Het 0.056, p-Egger 0.744.

Pooled estimates for allele and genotype distribution of the *TNF-A* 238G>A also had no significant association with the risk for HNP. No association was observed between genotype models and the risk of HNP: GG *vs.* GA+AA (OR: 1.60; 95%CI: 0.00, 12882.74), GA *vs.* GG+AA (OR: 0.63; 95%CI: 0.00, 4211.31), and AA *vs.* GG+GA (OR: 1.34; 95%CI: 0.16, 6712.96) ( Table 2 and Figure 3). Distribution of the allele also had no strong association with HNP susceptibility.

**Figure 4.**
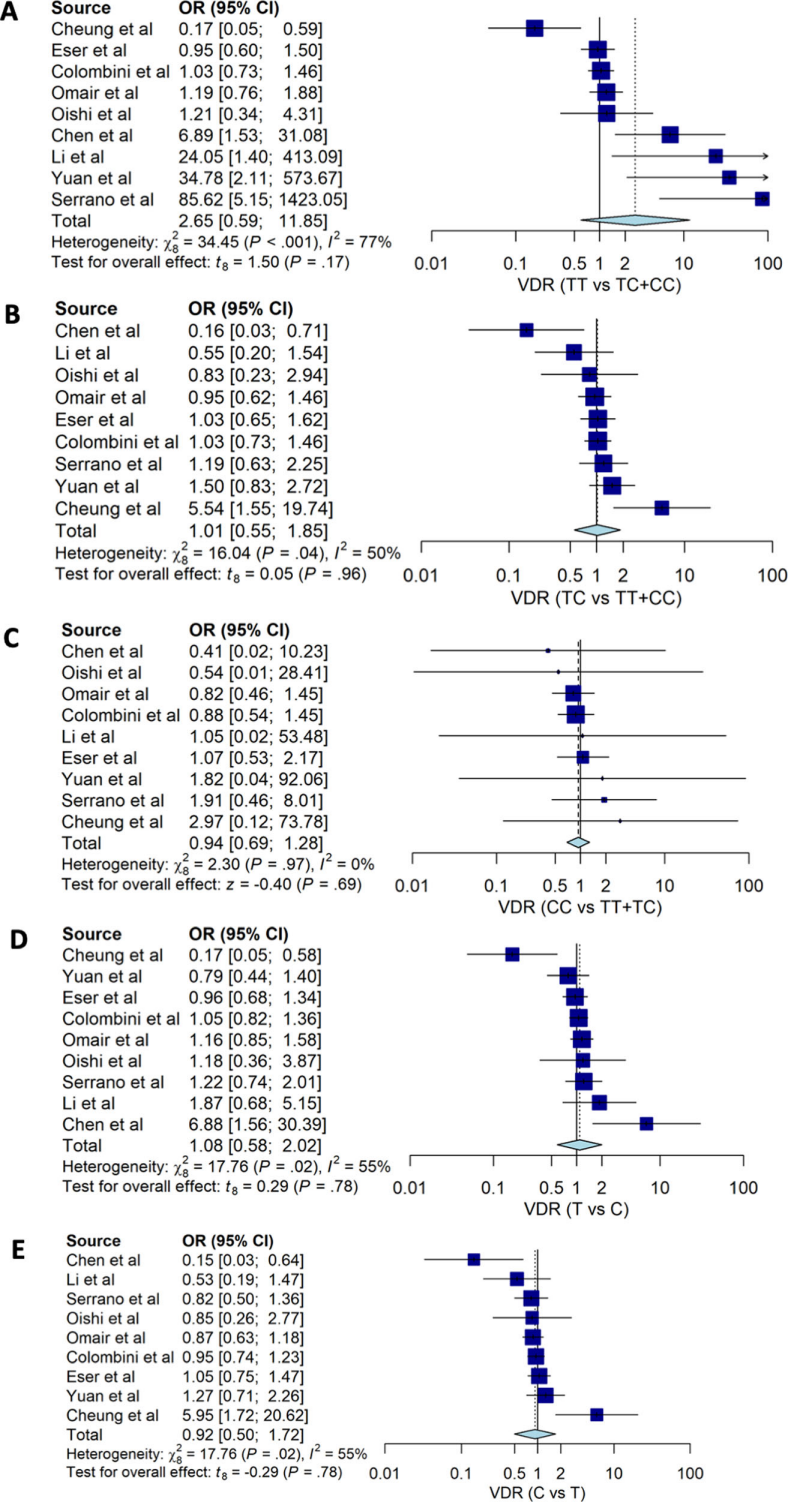
The forest plot of the association of *VDR TaqI* (rs731236) and HNP. (A) TT vs TC+CC (OR: 2.65; CI95%: 0.60, 11.85); p-value 0.172, p-Het <0.001, p-Egger 0.986, (B) TC vs TT+CC (OR1.01; CI95%: 0.55, 1.85); p-value 0.964, p-Het 0.041, p-Egger 0.791, (C) CC vs TC+TT (OR 0.94; CI95%: 0.69, 1.28); p-value 0.688, p-Het 0.970, p-Egger 0.387, (D) T vs C (OR: 1.06; CI95%: 0.91, 1.22); p-value 0.775, p-Het 0.023, p-Egger 0.874), (E) C vs T (OR: 0.92; 95%CI: 0.50, 1.72); p-value 0.775, p-Het 0.023, p-Egger 0.874.

Our estimates for genotypes of *VDR TaqI* (rs731236) suggested that none of the genotypes were associated with susceptibility to degenerative disc disease HNP with OR: 2.65; 95%CI: 0.60, 11.85 for TT *vs.* TC+CC, OR: 1.01; 95%CI: 0.55, 1.85 for TC vs. TT+CC and OR: 0.94; 95%CI: 0.69, 1.28 ( Table 2 and Figure 4). None of the alleles of *VDR TaqI* (rs731236) were associated with HNP; people with T allele had OD 1.06 with 95%CI: 0.91, 1.22 for HNP.

## Discussion

HNP occurs when the central part of the intervertebral disc, the nucleus pulposus, herniates through the surrounding part of the disc, the annulus fibrosus. The damage to the annulus fibrosus resulting in the herniated disc may be associated with factors such as gender, age, certain activities such as lifting of weights and carrying, and being overweight. ^52^ For example, the degeneration of disc organization could occur during aging as the regulation of the extracellular matrix (ECM), a major component of the disc, is damaged during the aging process. ^52^ The herniated disc or HNP may occur as a result of several pathological mechanisms. Those mechanisms ultimately cause imbalances in disc composition that are directly linked to the quality of the ECM. ^52^ Therefore, the balance between the ECM and its degrading enzymes, such as matrix metalloproteases (MMPs), seems to be the key to maintaining normal disc function. Interestingly, of several mechanical pathways, the regulation of the ECM and the MMPs could also be determined by the occurrence of single nucleotide polymorphisms in genes responsible for ECM regulation as explained below. ^53^

Two major structural proteins that are important in the matrix structure of the disc are collagen and proteoglycan. ^52^ The main proteoglycan found in the normal intervertebral disc is aggrecan. ^54^ Although both annulus fibrosus and nucleus pulposus are mainly composed of water, proteoglycan, and collagen, the level of those contents differ between the structures. The annulus fibrosus consists of 70% water, 15% collagen, and 5% proteoglycan, while the composition in the nucleus pulposus is 77% water, 4% collagen, and 14% proteoglycan. ^52^ Any event causing disturbances in those ratios and/or in synthesis and/or degradation of those structural proteins could lead to herniated disc problems. For example, hypoxic and acidic conditions could repress the synthesis of the matrix leading to the dysfunctionalities of the cells of the disc. ^55, 56^

The role of inflammation in predisposing the disc to damage has also been revealed. Specifically, proinflammatory cytokines are found to play a specific role in herniated and degenerated discs. ^54, 57- 59^ A controlled immunohistochemical study observed the accumulation of inflammatory cells, mainly macrophages, in herniated disc cells indicating the role of proinflammatory cytokines in the disease. ^58^ For example, IL-1β could induce the annulus fibrosus to generate inflammatory factors leading to the impairment of proteoglycan aggregation. ^59^ Another cytokine, TNF-α, is also involved in the development of intervertebral disc problems. ^60^ However, it seems that its effect is less significant than IL-1. ^61^ This finding may be related to its relatively lower expression compared to IL-1 in the normal and healthy disc. ^52, 62^

Penetration of those inflammatory cells or proteins could be caused by matrix loss. In normal conditions, aggrecan should prevent the penetration of various compounds, especially serum proteins and cytokines. ^63^ Therefore, in addition to its pivotal role in maintaining sufficient hydration to the disc, proteoglycan loss could stimulate the movement of cytokines towards the disc activating the inflammation cascade. ^52^

One of the mechanisms by which the proinflammatory cytokines, such as IL-1α and TNF-α, generate problems in the intervertebral disc is related to their effect on inducing MMP production. ^64- 67^ The exaggerated activity of MMPs causes excessive degradation of collagen and proteoglycan. ^54^ Another mechanism is associated with the activity of the cytokines in inhibiting tissue inhibitors of MMPs (TIMPs) which are responsible for terminating the action of MMPs. ^54^ Taken together, those actions ultimately impair disc functionality.

As the normal intervertebral disc is relatively avascular and aneural, ^52^ the nutritional supply to the disc depends on the ability of the nutrients to diffuse from the closest vascularized structure outside the disc which are the vertebral bodies. ^68^ The nutrients then penetrate the cartilaginous endplate and finally reach the annulus fibrosus and nucleus pulposus. ^68, 69^ Accordingly, calcification of the endplate would diminish diffusion of vital nutrients, leading to the death of the disc cells. ^70^ Therefore, VDR plays a critical role as this ligand-dependent transcription factor is involved in regulating calcium homeostasis and bone mineralization in the body, including in the intervertebral disc. ^71, 72^ It has been known that genetic polymorphisms occurring in genes encoding VDR are associated with intervertebral disc problems, ^72^ including herniated disc.

In conclusion, our results suggest that well-regulated IL-1A, TNF-A, and VDR are important for normal intervertebral discs and that dysregulation of these could negatively affect the intervertebral discs. Some individual studies found that SNPs in IL-1A (rs1800587), TNF-A (rs361525), and VDR (rs731236) were associated with the susceptibility to HNP, however, our meta-analysis suggested that the effects are not robust. There are some possible reasons for this finding. First, HNP is a complex disease and is cause by multiple factors. Thus, no single factor such as a single SNP is responsible for the whole pathogenesis. Second, large variations in the number of samples or allele frequencies among studies in our meta-analysis also contribute to the findings. This probably relates to differences in populations where study data were collected. Finally, the small number of samples significantly influenced the results of our meta-analysis. Therefore, studies with larger sample sizes and sub-analyses for different populations such as Asian, Caucasian, and other populations are warranted whenever more data are available.

## Data availability

### Underlying data

All data underlying the results are available as part of the article and no additional source data are required.

### Reporting guidelines

Figshare: PRISMA checklist for ‘The role of single nucleotide polymorphisms of IL-1A -889C>T (rs1800587), TNF-A -238G>A (rs361525), and VDR TaqI (rs731236) on susceptivity of herniated nucleus pulposus’, https://doi.org/10.7910/DVN/RBU2VR ^73^
