# Supplementary material for: The role of single nucleotide polymorphisms of IL-1A -889C>T (rs1800587), TNF-A -238G>A (rs361525), and VDR TaqI (rs731236) on susceptibility to herniated nucleus pulposus: a systematic review and meta-analysis
Source: F1000Res. 2021 Aug 23;10:419. Originally published 2021 May 25. [Version 3] doi: 10.12688/f1000research.53235.3 (PMC8406444; doi:10.12688/f1000research.53235.3)
Supplement: Peer review history

# Peer review: Reviewer response for version 3

**DOI:** 10.5256/f1000research.76428.r92543

**Published:** 2021-08-26

**Authors:** Ismail Hadisoebroto Dilogo

**Affiliations:** 1 Stem Cell Medical Technology Integrated Service Unit, Cipto Mangunkusumo Central Hospital, Faculty of Medicine, Universitas Indonesia, Jakarta, Indonesia; 2 Stem Cell and Tissue Engineering Research Cluster Indonesian Medical Education and Research Institute (IMERI), Universitas Indonesia, Jakarta, Indonesia; 3 Department of Orthopaedic and Traumatology, Dr. Cipto Mangunkusumo General Hospital, Faculty of Medicine, Universitas Indonesia, Jakarta, Indonesia

General remark:

- The title has been added and clarified with the study design “Systematic Review and Meta-Analysis” according to the previous comment.

Abstract:

- No further comment.

Introduction:

- No further comment.

Materials and Methods:

- No further comment.

Result and discussion:

- The discussion has been strengthened with comparison with previous studies that discussed about IL-1A, TNF-A, or VDR generally according to the previous comment.

Figures and Tables:

- No further comment.

Conclusion:

- No further comment.

Overall comments:

- This current manuscript version may be accepted for indexing.

Are the rationale for, and objectives of, the Systematic Review clearly stated?

Yes

Is the statistical analysis and its interpretation appropriate?

Partly

Are sufficient details of the methods and analysis provided to allow replication by others?

Yes

Are the conclusions drawn adequately supported by the results presented in the review?

Yes

Reviewer Expertise:

Stem cell and Tissue Engineering

I confirm that I have read this submission and believe that I have an appropriate level of expertise to confirm that it is of an acceptable scientific standard.

# Peer review: Reviewer response for version 2

**DOI:** 10.5256/f1000research.59208.r91376

**Published:** 2021-08-09

**Authors:** Ismail Hadisoebroto Dilogo

**Affiliations:** 1 Stem Cell Medical Technology Integrated Service Unit, Cipto Mangunkusumo Central Hospital, Faculty of Medicine, Universitas Indonesia, Jakarta, Indonesia; 2 Stem Cell and Tissue Engineering Research Cluster Indonesian Medical Education and Research Institute (IMERI), Universitas Indonesia, Jakarta, Indonesia; 3 Department of Orthopaedic and Traumatology, Dr. Cipto Mangunkusumo General Hospital, Faculty of Medicine, Universitas Indonesia, Jakarta, Indonesia

General remark:

- The study design on the title has not been added, I noticed that there is already a design label “Systematic Review” written above the title from the F1000 platform but since this manuscript will be added to some online databases such as Pubmed and Scopus, I recommend to put the design on the title. Also, please clarify if this is a meta-analysis or systematic review. As it is still written “Systematic review” above the title.

Abstract:

- The problem that this review wants to answer is added “this study was to determine the role of single nucleotide polymorphisms (SNPs) in interleukin 1 alpha ( *IL-1A*), tumor necrosis factor-alpha ( *TNF-A*), and vitamin D receptor ( *VDR*) genes on the susceptibility to herniated nucleus pulposus (HNP)” according to the previous comment.
- The study design of included samples in this study has been added according to the previous comment to show the level of evidence of this systematic review “three, two and nine case-control studies on *IL-1A *-889C>T, *TNF-A *-238G>A, and *VDR TaqI *were included, respectively, in our meta-analysis".
- For the conclusion, the author has stated the answer to the research question, although it is still inconclusive.

Introduction:

- No comment.

Materials and Methods:

- According to the previous comment, the author has clarified and stateD that there is no Randomized Controlled Trial and Cohort study about this topic in the limitation section.

Result and discussion:

- For the discussion, I noticed that author responded “To the best of our knowledge, our current study is the first study providing the holistic gene polymorphism in the case of HNP. Therefore, the comprehensive comparison in the context of methodological quality between our study and previous studies was unable to perform”. For comparing the result, you do not have to find the same method and result, please kindly compare it with similar or other previous studies that analyse similar variables. It doesn't have to be as specific as “IL-1A (rs1800587), TNF-A (rs361525), and VDR (rs731236)”. Please strengthen the discussion with comparison with previous studies that discussed IL-1A, TNF-A, or VDR as general.

Figures and Tables:

- No comment.

Conclusion

- The conclusion has been revised according to the previous comment, answering the research question. The conclusion is current existed studies are not strong enough to answer the question. Hence, further larger population and subgroup analyses are warranted to support these findings.

Are the rationale for, and objectives of, the Systematic Review clearly stated?

Yes

Is the statistical analysis and its interpretation appropriate?

Partly

Are sufficient details of the methods and analysis provided to allow replication by others?

Yes

Are the conclusions drawn adequately supported by the results presented in the review?

Yes

Reviewer Expertise:

Stem cell and Tissue Engineering

I confirm that I have read this submission and believe that I have an appropriate level of expertise to confirm that it is of an acceptable scientific standard, however I have significant reservations, as outlined above.

# Response

**Published:** 2021-08-10

**Authors:** Azharuddin Azharuddin

**Affiliations:** Universitas Syiah Kuala, Indonesia

General remark:

- The study design on the title has not been added, I noticed that there is already a design label “Systematic Review” written above the title from the F1000 platform but since this manuscript will be added to some online databases such as Pubmed and Scopus, I recommend to put the design on the title. Also, please clarify if this is a meta-analysis or systematic review. As it is still written “Systematic review” above the title.

RESPONSE: We have added the a "systematic review and meta-analysis" in our title. 

 Abstract:

- The problem that this review wants to answer is added “this study was to determine the role of single nucleotide polymorphisms (SNPs) in interleukin 1 alpha ( *IL-1A*), tumor necrosis factor-alpha ( *TNF-A*), and vitamin D receptor ( *VDR*) genes on the susceptibility to herniated nucleus pulposus (HNP)” according to the previous comment.

RESPONSE: Thank you. No comment. No further edit required. 

- The study design of included samples in this study has been added according to the previous comment to show the level of evidence of this systematic review “three, two and nine case-control studies on *IL-1A *-889C>T, *TNF-A *-238G>A, and *VDR TaqI *were included, respectively, in our meta-analysis".

RESPONSE: Thank you. No further edit required. 

- For the conclusion, the author has stated the answer to the research question, although it is still inconclusive.

RESPONSE: Thank you. No further edit required. 

 Introduction:

- No comment.

RESPONSE: No further edit required. 

 Materials and Methods:

- According to the previous comment, the author has clarified and stateD that there is no Randomized Controlled Trial and Cohort study about this topic in the limitation section.

RESPONSE: Thank you. No further edit required. 

 Result and discussion:

- For the discussion, I noticed that author responded “To the best of our knowledge, our current study is the first study providing the holistic gene polymorphism in the case of HNP. Therefore, the comprehensive comparison in the context of methodological quality between our study and previous studies was unable to perform”. For comparing the result, you do not have to find the same method and result, please kindly compare it with similar or other previous studies that analyse similar variables. It doesn't have to be as specific as “IL-1A (rs1800587), TNF-A (rs361525), and VDR (rs731236)”. Please strengthen the discussion with comparison with previous studies that discussed IL-1A, TNF-A, or VDR as general.

RESPONSE: Thank you for your suggestion. We have added some discussion based on previous studies. 

 Figures and Tables:

- No comment.

RESPONSE: Thank you. No further edit required. 

 Conclusion

- The conclusion has been revised according to the previous comment, answering the research question. The conclusion is current existed studies are not strong enough to answer the question. Hence, further larger population and subgroup analyses are warranted to support these findings.

RESPONSE: Thank you. No further edit required.

# Peer review: Reviewer response for version 1

**DOI:** 10.5256/f1000research.56597.r87698

**Published:** 2021-07-19

**Authors:** Mahir Gachabayov

**Affiliations:** 1 Section of Colorectal Surgery, Department of Surgery, Westchester Medical Center, New York Medical College, Valhalla, NY, USA

Thank you for the opportunity of reviewing this manuscript. 

- This is a systematic review and meta-analysis aiming at evaluating the impact of SNPs on susceptibility to HNP. 
- The research question makes sense. The gap in the literature was well presented and the objective of the review was well formulated.
- The protocol of the systematic review was prospectively developed and registered in PROSPERO. 
- The search strategy was comprehensive.The screening and study selection process were well described and illustrated in a PRISMA flow diagram. Methodology of quality assessment and statistical analysis was adequate.
- The results are well presented, summarized in tables, and illustrated in forest plots. 
- The Discussion is comprehensible, well-describes the findings of this systematic review in comparison with the current body of evidence.
- Conclusions are justified by the findings.

I have one minor comment:

- It seems like the limitations were presented in the conclusion. I would separate them from the conclusions and move them as the last paragraph of the Discussion.

Are the rationale for, and objectives of, the Systematic Review clearly stated?

Yes

Is the statistical analysis and its interpretation appropriate?

Yes

Are sufficient details of the methods and analysis provided to allow replication by others?

Yes

Are the conclusions drawn adequately supported by the results presented in the review?

Yes

Reviewer Expertise:

Clinical outcomes research and evidence synthesis

I confirm that I have read this submission and believe that I have an appropriate level of expertise to confirm that it is of an acceptable scientific standard.

# Response

**Published:** 2021-08-03

**Authors:** Azharuddin Azharuddin

**Affiliations:** Universitas Syiah Kuala, Indonesia

**#Reviewer 2**

*I have one minor comment: It seems like the limitations were presented in the conclusion. I would separate them from the conclusions and move them as the last paragraph of the Discussion.*

**Response:** The limitation has been provided in the end of discussion section. The conclusion has been revised as suggested.

# Peer review: Reviewer response for version 1

**DOI:** 10.5256/f1000research.56597.r86114

**Published:** 2021-06-01

**Authors:** Ismail Hadisoebroto Dilogo

**Affiliations:** 1 Stem Cell Medical Technology Integrated Service Unit, Cipto Mangunkusumo Central Hospital, Faculty of Medicine, Universitas Indonesia, Jakarta, Indonesia; 2 Stem Cell and Tissue Engineering Research Cluster Indonesian Medical Education and Research Institute (IMERI), Universitas Indonesia, Jakarta, Indonesia; 3 Department of Orthopaedic and Traumatology, Dr. Cipto Mangunkusumo General Hospital, Faculty of Medicine, Universitas Indonesia, Jakarta, Indonesia

General remark:

- The title is descriptive about the manuscript, however, please state the study design on the title.
- To draw the interest of the reader, is it possible if the authors simplified the title from “IL-1A-889C>T(rs1800587)" to “IL-1A”, and also the same for TNF-A and VDR?

 Abstract:

- Line 31, please state the problem that this review will answer.
- Line 35, in the methods section, please state the study design of included samples in this study to show the level of evidence of this meta-analysis.
- Line 58, for the conclusion, please kindly state the answer and summary of the research question, instead of restating limitation and result.

Introduction:

- The Introduction is already well written. It gives enough information for the reader to understand the context. It also clearly explains why the study was necessary.

Materials and Methods:

- As all the studies included in this review are a case-control, though it is uncommon for a systematic review meta-analysis to use a case control as a sample, please clarify and state that there is no Randomized Controlled Trial and Cohort study about this topic.
- I noticed the authors used Newcastle Ottawa Score, please kindly check https://ebm.bmj.com/content/23/2/60 to review the methodological quality of a case series.
- Please define the primary outcome in this review.

Result and discussion:

- The results are presented clearly and accessibly, and it also supports the main conclusion.
- The discussion, has to be more detailed, from line 203- 244, the discussion mainly discusses the pathogenesis of the herniated nucleus pulposus. Please kindly deepen the discussion based on the result of this review, compare it with previous study(s), and explain the finding from the authors' point of view.

Figures and Tables:

- Figures and Tables are adequately explained.

Conclusion:

- The limitations are well stated, however, line 286-288 “Therefore, studies with larger sample sizes and sub-analyses for different populations such as Asian, Caucasian, and other populations are warranted whenever more data are available.” Please put in one paragraph before the conclusion to make the conclusion from your review stronger.

Overall comments:

- This manuscript may be considered for indexing after Major revisions.

Are the rationale for, and objectives of, the Systematic Review clearly stated?

Yes

Is the statistical analysis and its interpretation appropriate?

Partly

Are sufficient details of the methods and analysis provided to allow replication by others?

Yes

Are the conclusions drawn adequately supported by the results presented in the review?

Yes

Reviewer Expertise:

Stem cell and Tissue Engineering

I confirm that I have read this submission and believe that I have an appropriate level of expertise to confirm that it is of an acceptable scientific standard, however I have significant reservations, as outlined above.

# Response

**Published:** 2021-08-03

**Authors:** Azharuddin Azharuddin

**Affiliations:** Universitas Syiah Kuala, Indonesia

**#Reviewer 1**

*Abstract: Line 31, please state the problem that this review will answer.*

**Response:** We have added the problem that underly our study in abstract: “The pathogenesis of herniated nucleus pulposus (HNP) is complex, and may involve the wide variety of gene polymorphism. However, the reports from the existing evidence revealed inconclusive findings.”

*Abstract: Line 35, in the methods section, please state the study design of included samples in this study to show the level of evidence of this meta-analysis.*

**Response:** the study design of included articles has been added in the results section of abstract: “We screened 3,067 unique studies for eligibility and three, two and nine case-control studies on IL-1A -889C>T, TNF-A -238G>A, and VDR TaqI were included, respectively, in our meta-analysis.”

*Abstract: Line 58, for the conclusion, please kindly state the answer and summary of the research question, instead of restating limitation and result.*

**Response:** The main findings of our study has been outlined in the conclusion section of abstract: “our data indicated that IL-1A -889C>T, TNF-A -238G>A, and VDR TaqI had weak association with HNP susceptibility in both genotypes and allele distributions.” However, we also described about the clinical implication, limitation and future research direction to prevent the miss-understanding in interpretating the findings of our study.

*Introduction: The Introduction is already well written. It gives enough information for the reader to understand the context. It also clearly explains why the study was necessary.*

**Response:** thank you.

*Materials and Methods: As all the studies included in this review are a case-control, though it is uncommon for a systematic review meta-analysis to use a case control as a sample, please clarify and state that there is no Randomized Controlled Trial and Cohort study about this topic.*

**Response: **We agree that case-control studies are uncommon to include in meta-analysis. However, in the meta-analysis, we cannot manage the findings (the findings of article searching) as we expected if they are not supported by the available data. And unfortunately, in the searching strategy, we only found case-control studies. This point has also been included in the limitations of our study.

*Materials and Methods: I noticed the authors used Newcastle Ottawa Score, please kindly check https://ebm.bmj.com/content/23/2/60 to review the methodological quality of a case series.*

**Response:** We thank you for the suggestion. Case report and or case series studies may be possible to include in systematic review. However, they are not possible  to include in meta-analysis (double arm calculation, as reported in our current study) because they do not have control.

*Materials and Methods: Please define the primary outcome in this review.*

**Response: **The primary outcomes have been provided in the Method section: “Covariates and sub-group analysis. The outcome measure in our study was the incidence of HNP while the predictor covariates were the gene polymorphisms of IL-1A (rs1800587), TNF-A (rs361525), and VDR (rs731236). All genetic models were applied to describe the role of each gene variant in the pathogenesis of HNP. For IL-1A (rs1800587), the allele models were C vs. T and T vs. C; and the genotype models were CC vs. CT + TT, CT vs. CC + TT, and TT vs. CC + CT. For TNF-A (rs361525), the allele models were G vs. A and A vs. G; and the genotype models were GG vs. GA + AA, GA vs. GG + AA, and AA vs. GG + GA. For VDR (rs731236), the allele models were T vs. C and C vs. T; and the genotype models were TT vs. TC + CC, TC vs. TT + CC, and CC vs. TT + TC.”

*Result and discussion: The results are presented clearly and accessibly, and it also supports the main conclusion.*

**Response:** Thank you.

*The discussion, has to be more detailed, from line 203- 244, the discussion mainly discusses the pathogenesis of the herniated nucleus pulposus. Please kindly deepen the discussion based on the result of this review, compare it with previous study(s), and explain the finding from the authors' point of view.*

**Response: **The explanation on our main findings and the holistic comparison including the possible reason of our findings has been provided: “Our present study failed to clarify the role of IL-1A (rs1800587), TNF-A (rs361525), and VDR (rs731236) in the pathogenesis of HNP. To the best of our knowledge, our current study is the first study providing the holistic gene polymorphism in the case of HNP. Therefore, the comprehensive comparison in the context of methodological quality between our study and previous studies was unable to perform. However, several possible reason for the negative findings in our study might be proposed. First, HNP is a complex disease and is cause by multiple factors. Thus, no single factor such as a single SNP is responsible for the whole pathogenesis. Second, large variations in the number of samples or allele frequencies among studies in our meta-analysis also contribute to the findings. This probably relates to differences in populations where study data were collected. Finally, the small number of samples significantly influenced the results of our meta-analysis. Therefore, studies with larger sample sizes and sub-analyses for different populations such as Asian, Caucasian, and other populations are warranted whenever more data are available.”

*Figures and Tables: Figures and Tables are adequately explained.*

**Response:** Thank you.

*Conclusion: The limitations are well stated, however, line 286-288 “Therefore, studies with larger sample sizes and sub-analyses for different populations such as Asian, Caucasian, and other populations are warranted whenever more data are available.” Please put in one paragraph before the conclusion to make the conclusion from your review stronger.*

**Response:** The conclusion has been revised as suggested.

*Overall comments: This manuscript may be considered for indexing after Major revisions.*

**Response: **Thank you.
